# Quality of Life of Patients with Mandibular Third Molars and Mild Pericoronitis. A Comparison between Two Different Treatments: Extraction or Periodontal Approach

**DOI:** 10.3390/antibiotics9050222

**Published:** 2020-04-29

**Authors:** P. C. Passarelli, M. A. Lopez, V. Desantis, G. B. Piccirillo, E. Rella, V. Giovannini, A. Speranza, M. De Leonardis, P. F. Manicone, M. Casale, A. D’Addona

**Affiliations:** 1 Department of Head and Neck, Division of Oral Surgery and Implantology, Institute of Clinical Dentistry, Catholic University of the Sacred Heart, Gemelli University Polyclinic Foundation, 00168 Rome, Italy; desantisviviana@gmail.com (V.D.); giovanbpiccirillo@gmail.com (G.B.P.); rellaedoardo@gmail.com (E.R.); valerio.giovannini01@gmail.com (V.G.); alesperanza121@gmail.com (A.S.); marta_de_leo@hotmail.it (M.D.L.); paolofrancesco.manicone@policlinicogemelli.it (P.F.M.); antonio.daddona@policlinicogemelli.it (A.D.); 2 Unit of Otolaryngology, University Campus Bio-Medico, 00128 Rome, Italy; micheleantonio.lopez@gmail.com (M.A.L.); m.casale@unicampus.it (M.C.)

**Keywords:** quality of life, mandibular third molar, tooth extraction, oral surgery

## Abstract

**Background:** The extraction of the mandibular third molar is one of the most frequent intervention in oral surgery. A common indication for wisdom tooth extraction is represented by pericoronitis, which can determine discomfort and pain in patients. The present study aimed to evaluate the impact of patients’ quality of life by comparing a surgical approach with a periodontal approach. Methods: We evaluated 82 patients diagnosed with pericoronitis that occurred at the third molar site. In total, 41 of them received a periodontal treatment and 41 were treated by extraction. The quality of life (QoL) of the patients was assessed by using the Oral Health Impact Profile-14 (OHIP-14) index. Results: A total of 82 patients were included in the study and were followed up for 6 months. Of the patients, 41 received a periodontal treatment and 41 underwent surgical extraction. At the baseline, the OHIP-14 scores of the surgical group were higher (19.71, SD 9.90) than the periodontal group (14.41, SD 8.71). At 1 week, there was a reduction in terms of OHIP-14 in both groups, but the periodontal group showed lower values (12.3, SD 8.11). Long-term follow-up showed a reduction of the OHIP-14 values, with a difference in favor of the surgical group (0.10, SD 0.45). However, there was a reduction in OHIP-14 scores in both groups. Conclusion: Although the periodontal treatment offered a rapid improvement in terms of quality of life during the first week after the treatment, after 1 month and 6 months, the extraction of the mandibular third molar extraction remained the best treatment, removing the occurrence of re-inflammation of the site.

## 1. Introduction

Pericoronitis is defined as an acute or chronic periodontal inflammation of the soft tissue around the crown of an impacted or semi-impacted tooth [1]; it usually affects the lower third molars.

The main symptoms can include dysphagia, trismus, and purulence, but also swelling, pain, and fever [2].

The extraction of the mandibular third molar is one of the most frequent interventions in oral surgery [3], and the occurrence of pericoronitis is considered the most frequent indication for the extraction of these teeth [4]; moreover, an asymptomatic third molar can lead to a periodontal damage on the second molar [5].

Clinicians may have to choose between two treatment options, which lead to different related consequences—after the extraction of the third molar, the alveolar ridge undergoes a progressive bone resorption [6], whereas, on the other hand, the periodontal treatment is not a definitive solution for pericoronitis.

Over recent years, health-related outcomes have become important, such as the impact of a treatment on daily living and patient satisfaction.

The concept of quality of life (QoL) has been defined by the World Health Organization as “an individual perception of their position in file in the context of the culture and value system in which they live and in relation to their goals, expectations, standards and concerns” [7].

The most used index is the Oral Health Impact Profile (OHIP)-49 and its short version OHIP-14 [8].

Several studies in the literature showed that pericoronitis can cause discomfort to the patient, and that the situation can be improved with the extraction of those elements [2,9].

This study aimed to compare how quality of life may change depending on whether the treatment is the extraction or a periodontal approach, during the six months following treatment.

## 2. Results

A total of 82 patients met the inclusion criteria—45 (54.8%) were women and 37 (45.2%) men.

The mean age was 42.4 years in the group of patients treated with surgical extraction and 37.7 years in the patients who underwent periodontal therapy; age and sex differences between the two groups are reported in Table 1. The patients were equally divided between the two groups, with 41 patients receiving a periodontal treatment and 41 patients undergoing surgical extraction. 

We can assume that both therapies determined a significant improvement in the quality of life of the patients and we had different OHIP-14 scores—there was a reduction of this index between the baseline, the first month, and the sixth month (Table 2).

At the baseline, both groups revealed high OHIP-14 scores, but the surgically treated group showed the worst outcomes in terms of quality of life, with higher OHIP-14 outcomes (19.71, SD 9.90) than the periodontal group (14.41, SD 8.71). At 1 week, there was a reduction in of OHIP-14 in both groups, but the periodontal group showed lower values (12.3, SD 8.11). Long-term follow-up at 1 month and 6 months showed a reduction in terms of OHIP-14 values, with a difference in favor of the surgical group (0.10, SD 0.45). However, there was a reduction a significant improvement of the quality of life in both groups.

## 3. Materials and Methods 

This study included 82 consecutive patients who underwent third molar extraction over a period from March 2018 to March 2019 at the Oral Surgery Unit, Policlinico Universitario Agostino Gemelli (Rome, Italy).

Because of the retrospective nature of the present study, it was granted an exemption in writing by the institutional review board of the Catholic University of Sacred Heart of Rome. It was conducted in accordance to the requirements of the Declaration of Helsinki.

The inclusion criteria were patients with good general health (American Society of Anesthesiologists I or II), aged between 18 and 75, the presence of mild clinical signs of pericoronitis, and a good periodontal status by the standard set by the European Federation of Periodontology/American Academy of Periodontology [10].

We evaluated systemic disease [11], syndromic diseases [12] that can cause dental abnormalities and immune disorders [13], in order to achieve a better management of the relative contraindications to the extractions and also to avoid absolute contraindications.

It is very important to assess the general health situation of the patients and to evaluate the presence of bleeding disorders, which can make it difficult to manage post-operative complications, unless a close collaboration between the dentist and the hematologists is established [14,15].

Patients with major symptoms of pericoronitis were excluded.

All subjects provided written informed consent, and QoL data were collected for each patient by using the OHIP-14 index.

All the 41 lower molar extractions were performed by the same practitioner, whereas another examiner performed the periodontal treatment.

An orthopantomography X-ray image was obtained and discussed before the surgical procedures to assess the position of the third molar; we used X-ray images obtained with a digital method, as they show a better diagnostic precision [16]. 

The most used system to indicate the depth of third molars impaction is the classification by Pell and Gregory, whereas to evaluate the angle, the preferred criterion was the classification of Winter.

We found that pericoronitis is mainly associated with vertical positioned third molar (according to Winter classification) and with class I or II, type A, according to Pell and Gregory classification, and we included in our study patients who presented these kinds of third molars impaction.

Check-up and follow-up appointments were taken after 1 week, 1 month, and 6 months, and QoL data were collected again at each appointment.

Two weeks before tooth extraction, the patients underwent periodontal charting and scaling procedures to establish proper oral hygiene conditions [17], and oral hygiene instructions were given. Patients who received a non-surgical treatment for the pericoronits instead underwent a full mouth scaling and a deep scaling and root planning, and the patients were instructed to rinse their mouth twice a day for 1 week with 0.2% chlorhexidine mouthwash.

We collected all the data from questionnaires at the baseline, at the first month, and at the third month after treatment.

The data were analyzed using the IBM SPSS Statistics (Version 20, IBM Corp) software package. The Mann–Whitney *U* test and Friedman’s tests were used to assess the significance of differences. Probabilities <0.05 were accepted as significant.

## 4. Conclusions

During the second half of the last century, the extraction of an impacted third molar has become a very common and a routine procedure [18] in dental practice. The aim of this study was to compare the impact on the patients’ quality of life by comparing a surgical and a non-surgical conservative approach.

The outcomes of the present study showed that a permanent improvement of the quality of life was reached with the extraction of the third molar; on the other hand, a periodontal treatment of symptomatic third molars can lead to a temporary pain relief, but there may be the risk of further re-inflammation of the site.

In those patients who underwent tooth extraction, pain and discomfort were present during the first week; in fact, post-operative complications may have an adverse effect on the quality of life of the patients [19]. On the other side, patients who received the periodontal treatment showed immediate pain relief. During the first week following treatment, patients treated with a non-surgical approach showed less symptoms. Follow-up at 1 month and at 6 months demonstrated instead that there was a slight improvement in the quality of life of patients undergoing third molar extraction. Thus, a faster improvement in terms of QoL was present in patients who received a periodontal treatment, but tooth extraction revealed to be more successful after long-term follow-up.

A limitation of this study is the subjectivity of the data and the perception of the QoL, which varied between patients. Dentists cannot establish that pain is the most important symptom and the pain cannot be a direct symptom in the choice of treatment—it is important to understand the other factors that may affect QoL. Moreover, it can be useful to have a long-term follow-up because there could be late complications [20].

Moreover, patients treated with a periodontal treatment may develop recurrent inflammation, and thus we can conclude that a surgical approach is more effective than the periodontal treatment.

Further long-term studies are required in this subject to determine the most appropriate approach for the patient. 

## Figures and Tables

**Table 1:** Patients characteristics.

Patient Details	Periodontal Treatment *N* = 41	Surgical Treatment *N* = 41	*p*-Value
**Sex**			0.01 *
Females	24	13	
Males	17	28	
**Age**, mean (SD)	37.7 (3.97)	42.4 (4.14)	0.001 **

* Chi^2^ test; ** Student’s *t*-test.

**Table 2:** Oral Health Impact Profile (OHIP)-14 scores in the periodontal and surgical groups at baseline, and postoperatively at the first week, the first month, and at 6 months.

Variable	Periodontal Treatment *N* = 41	Surgical Treatment *N* = 41	*p*-Value *
Baseline (a)	14.41 (8.71)	19.71 (9.90)	**0.013**
First week (b)	12.3 (8.11)	18.45 (9.72)	**0.015**
First month (c)	1.3 (1.16)	0.42 (0.57)	**0.04**
Sixth month (d)	0.14 (0.37)	0.10 (0.45)	0.39
*p*-Value **	<0.0001	<0.0001	−

* Mann–Whitney *U* test; ** Friedman’s test.
